# Death of Professor Dieffenbach

**Published:** 1848-04

**Authors:** 


*Death of the celebrated Professor Dieffenbach.*—We announced in a former number the death of this eminent Surgeon. For the following biographical sketch we are indebted to the London Lancet:

A private correspondent in Berlin informs us that this distinguished surgeon dropped down dead, at three o'clock in the day, on the 11th instant, just after lecturing. He had previously been in good health. The career of this talented and eccentric character might form the subject for a romance. J. F. Dieffenbach was a theological student in one of the German universities, in 1812. At the time of the war of liberation he entered the Prussian service, and fought as a private soldier at the battle of Waterloo. After the peace he took to the profession of the law; but from some disagreement with the professor of the university in which he studied, he relinquished that pursuit, and studied medicine. Great were his difficulties, and long his early struggles; at last he came to Berlin, and there his unwearied energy, his boldness, and thorough determination to succed, finally met its reward. Dieffenbach labored among the poor, and his first rise is owingto the following curious circumstance:—Rust, the celebrated surgeon and anatomist, and at that time proto-medicus of Prussia, one night missed his way on leaving the apartment of a patient, and coming down by a back stairs in one of the great houses or hotels in Berlin, was attracted by a light which proceeded from a small chamber, not larger than a dog kennel, at the foot of the stairs. On looking in, he was not a little astonished to see the apartment occupied by two old women and a young man, who by the glimmer of a small flickering lamp, was operating for a strangulated hernia, on one of the crones, who lay upon a wretched pallet—the operator was Dieffenbach. This was the beginning of his good fortune. Surgery has lost one of the boldest operators in Europe, and the charité clinique its chief attraction: he might have been with justice styled the father of plastic surgery. His improvements in this branch—his operations for the removal of squint, and his various ingenious contrivances for the removal of deformities, and the restoration of lost parts, are already well known. The cause of his sudden death had not been ascertained when our correspondent wrote. He was between fifty-five and sixty years of age.

